# Bone Morphogenetic Proteins and myostatin pathways: key mediator of human sarcopenia

**DOI:** 10.1186/s12967-017-1143-6

**Published:** 2017-02-15

**Authors:** Manuel Scimeca, Eleonora Piccirilli, Francesca Mastrangeli, Cecilia Rao, Maurizio Feola, Augusto Orlandi, Elena Gasbarra, Elena Bonanno, Umberto Tarantino

**Affiliations:** 1 Multidisciplinary Study of the Effects of Microgravity on Bone Cells” Project, Spatial Biomedicine Center, Italian Space Agency (ASI), Via del Politecnico snc, 00133 Rome, Italy; 2 Anatomic Pathology Section, Department of Experimental Medicine and Surgery, University of Rome ‘Tor Vergata’, Via Montpellier 1, 00133 Rome, Italy; 3 Department of Orthopedics and Traumatology, “Policlinico Tor Vergata” Foundation, Viale Oxford 1, 00133 Rome, Italy; 4 Department of Biomedicine and Prevention, University of Rome ‘Tor Vergata’, Via Montpellier 1, 00133 Rome, Italy; 5 Department of Clinical Sciences and Translational Medicine, University of Rome Tor Vergata, Via Montpellier 1, 00133 Rome, Italy

## Abstract

**Background:**

Sarcopenia, osteoporosis and osteoarthritis are the most frequent musculoskeletal disorders affecting older people. The main aim of this study was to test the hypothesis that the balance between BMPs and myostatin pathways regulates the age-related muscle degeneration in OP and OA patients. To this end, we investigated the relationship among the expression of BMP-2/4-7, myostatin and phosphorylated Smads1-5-8 and the muscle quality, evaluated in term of fibers atrophy and satellite cells activity.

**Methods:**

In this retrospective study, we collected 123 biopsies of vastus lateralis: 48 biopsies from patients who underwent hip arthroplasty for subcapital fractures of the femur (OP), 55 biopsies from patients who underwent hip arthroplasty for osteoarthritis (OA) and 20 biopsies from patients who underwent hip arthroplasty for high-energy hip fractures (CTRL). Muscle biopsies were fixed in 4% paraformaldehyde and paraffin embedded. Serial sections were used for morphometrical and immunohistochemical analysis (BMP/2/4-7, myostatin, Smads1-5-8, Pax7 and myogenin). In addition, 1 mm^3^ of muscle tissue of each patient was embedded in epon for ultrastructural study.

**Results:**

Morphometric data indicated an increase of the number of atrophic fibers in OP patients compare to OA. In line with these data, we found an high regenerative potential in muscle tissues of OA patients due to the significant amount of both Pax7 and myogenin positive satellite cells detected in OA group. In addition, our data showed the decrease of BMP2/4 and -7 expression in OP patients compared to both OA group and CTRL. Conversely, OP patients were characterized by high levels of myostatin expression. A different expression profile was also found for phosphorylated Smad1-5-8 between OP and OA patients. In particular, OP patients showed a low number of positive phosphorylated Smad1-5-8 nuclei.

**Conclusion:**

The identification of molecular pathways involved in the pathogenesis of sarcopenia open new prospective for the development of drugs able to prevent/treat the muscle impairment that occur in elderly. Results here reported, highlighting the role of BMPs and myostatin pathways in physio-pathogenesis of human sarcopenia, allow us to propose human recombinant BMP-2/7 and anti-myostatin antibodies as a possible therapeutic option for the sarcopenia.

## Background

Sarcopenia is an aging-induced generalized pathological condition characterized by loss of muscle mass and function related to aging [1, 2]. It is strongly associated to reduction of the global physical strength and poor quality of life¸ ultimately the patient experiences fall and fractures and is confined to bed with an increased risk of mortality [3]. Osteoporosis (OP), osteoarthritis (OA) and sarcopenia are the most frequent musculoskeletal disorders affecting older people [4, 5]. Indeed, aging process is a factor involved in the loss of the functionality of both bone and muscle [6, 7]. In this context, emerging evidence suggests that Bone Morphogenetic Proteins (BMPs) may play an important role in both muscle and bone homeostasis [8]. The BMPs are molecules of transforming growth factor-β (TGF-β) family that orchestrates various biological processes linked to cell proliferation, differentiation, morphogenesis, cell homeostasis and regeneration [9]. Recently, we and others groups have shown that the BMPs expression has a role in controlling adult skeletal muscle mass and regeneration [10–12]. In particular, we found an association between BMP-2 and BMP-4 expression and the activity of satellite stem cells [13]. Among BMPs family have been identified numerous molecules with positive and/or negative effects on muscle cells [8]. As concern BMP-7, recent studies demonstrated their ability to block/reduce muscle atrophy after denervation [14]. In the canonical signaling pathway, they initiate the signal transduction cascade by binding BMP-receptors and activating Smad (small mother against decapentaplegic) proteins. The Smads involved in BMP signaling are Smad1, Smad5, and Smad8 (Smad1/5/8) [15]. Activated Smads then associate with the Smad4, and translocates to the nucleus where it functions as a transcription factor regulating the expression of gene involved in muscle homeostasis, such as MyoD [16]. Myostatin is a member of the TGF-β superfamily and acts as a potent negative regulator of skeletal muscle growth [17]. It is known to affect muscle mass by negative regulation of myogenesis [18]. Indeed, in vitro experiments have shown that myostatin blocks myoblast proliferation and satellite cell proliferation and self-renewal by down regulation of MyoD [19]. Myostatin induced the blocks of muscle regeneration competing both for the binding with BMP-receptor and activation of Smad4. Thus, the balance between myostatin and BMP signaling strongly influence the muscle quality.

The main aim of this study was to test the hypothesis that the balance between BMPs and myostatin pathways regulates the age-related muscle degeneration in OP and OA patients. To this end, we investigated the relationship among the expression of BMP-2/4-7, myostatin and phosphorylated Smads1-5-8 and the muscle quality, evaluated in term of fibers atrophy and satellite cells activity.

## Methods

### Patients

We enrolled 123 patients who underwent hip surgery in the Orthopedic Department of “Tor Vergata” University Hospital in the period June 2014–February 2015. We enrolled 48 consecutive patients who underwent hip arthroplasty for subcapital fractures of the femur (35 women and 13 men), 55 consecutive patients who underwent hip arthroplasty for osteoarthritis (40 women and 15 men and 20 consecutive patients under 50 (8 women and 12 men) who underwent hip arthroplasty for high-energy hip fractures (CTRL). Exclusion criteria were history of cancer, myopathies or other neuromuscular diseases or chronic administration of corticosteroid for autoimmune diseases (more than 1 month), diabetes, alcohol abuse, and HBV, HCV, or HIV infections.

### Bone mineral density evaluation (DXA)

DXA was performed with a Lunar DXA apparatus (GE Healthcare, Madison, WI, USA). Lumbar spine (L1–L4) and femoral (neck and total) scans were performed, and bone mineral density (BMD) was measured according to manufacture recommendations [20]. Dual-energy X-ray absorptiometry measures BMD (in grams per square centimeter), with a coefficient of variation of 0.7%. For patients with fragility fractures, BMD was measured on the uninjured limb. For all the other patients, measurements were performed on the non-dominant side, with the participants supine on an examination table with their limbs slightly abducted [21]. DXA exam was performed 1 day before surgery for osteoarthritis patients (OA), and 1 months after surgery for osteoporotic (OP) and CTRL patients. The results were expressed as T-scores.

### Radiological evaluation

Hip X-rays were performed in order to check the fracture or to assess hip osteoarthritis. Kellgren–Lawrence scale was used in order to determine the severity of osteoarthritis. The Kellgren and Lawrence system is a method of classifying the severity of osteoarthritis (OA) using five grades. This classification was proposed by Kellgren et al. in 1957 [22]. It includes: grade 0 if no radiographic features of OA are present; grade 1 if doubtful joint space narrowing (JSN) and possible osteophytic lipping; grade 2 if definite osteophytes and possible JSN on anteroposterior weight-bearing radiograph; grade 3 if multiple osteophytes, definite JSN, sclerosis, possible bony deformity; grade 4 if large osteophytes, marked JSN, severe sclerosis and definite bony deformity. Two orthopedists independently assessed all radiographs. Patients with a grade of K –L  ≥  2 were considered osteoarthritic.

### Sampling

During open surgery for hip arthroplasty muscle biopsies were taken from the upper portion of the vastus lateralis. Sample withdrawals were performed for histological analysis excluding macroscopic alteration of skeletal muscle biopsy as necrosis areas.

### Histology

Muscle biopsies were fixed in 4% paraformaldehyde for 24 h and paraffin embedded. Three-micrometer thick sections were stained with hematoxylin and eosin (H&E) and the histological evaluation blindly was performed by two pathologists.

### Morphometric analysis

In order to assess fibers atrophy, a minimum of 250 muscle fibers per biopsy have been evaluated, comparing minimum transverse diameter and cross-sectional area of type I and type II fibers for relative prevalence. A threshold diameter lower than 30 μm (minimum value of the normal range for women) characterized atrophic fibers.

### Immunohistochemistry

BMP-2, BMP-4, BMP-7, myostatin, phosphorylated Smads1-5-8, Pax7 and myogenin expression were assed in muscle biopsies by immunohistochemistry. Briefly, antigen retrieval was performed on 4-μm-thick paraffin sections using EDTA citrate pH 7.8 or Citrate pH 6.0 buffers for 30 min at 95 °C. Sections were then incubated for 1 h at room temperature with primary antibodies (listed in Table 1). Washings were performed with PBS/Tween20 pH 7.6. Reactions were revealed by HRP–DAB Detection Kit (UCS Diagnostic, Rome, Italy). Immunohistochemistry was evaluated by two blind observers by counting the number of positive fibers (out of a total of 500 in randomly selected regions) for BMP-2, BMP-4, myiostatin and Smads reaction, and by counting the number of positive satellite cells for Pax7 and myogenin expression. To assess the background of immuno-staining, for each reaction, we included a negative control, incubating the sections with secondary antibodies (HRP) and a detection system (DAB).

**Table 1:** Primary antibodies used for immunohystochemical reactions

Antibody	Characteristics	Dilution	Retrieval
Anti-Pax7	Rabbit monoclonal, clone NC, Novus Biologicals	1:100	Citrate pH 6.0
Anti-myogenin	Rabbit monoclonal, clone F5D, AbCam Cambridge, UK	1:100	EDTA citrate pH 8.0
Anti-BMP-2	Rabbit clone N/A; Novus Biologicals, Littleton, CO, USA	1:100	Citrate pH 6.0
Anti-BMP-4	Rabbit polyclonal clone NBP1-91805; Novus Biologicals, Littleton, CO, USA	1:500	Citrate pH 6.0
Anti-BMP-7	Mouse monoclonal clone ab54904; AbCam, Cambridge, UK	1:250	Citrate pH 6.0
Anti-myostatin	Rabbit monoclonal, clone ab134682, AbCam, Cambridge, UK	1:100	EDTA citrate pH 8.0
Anti-Smad1-5-8	Rabbit polyclonal, clone ab13723, AbCam, Cambridge, UK	1:100	EDTA citrate pH 8.0

### Transmission electron microscopy (TEM)

One cubic millimetre of muscle tissue from surgical specimens were fixed in 4% paraformaldehyde and post-fixed in 2% osmium tetroxide [23]. After washing with 0.1 M phosphate buffer, the sample was dehydrated by a series of incubations in 30, 50 and 70% ethanol. Dehydration was continued by incubation steps in 95% ethanol, absolute ethanol and propylene oxide, after which samples were embedded in Epon (Agar Scientific, Stansted, Essex CM24 8GF United Kingdom) [24]. Eighty µm ultra-thin sections were mounted on copper grids and examined with a transmission electron microscope (Model 7100FA, Hitachi, Schaumburg, IL, USA).

### Statistical analysis

Statistical analysis was performed using GraphPad Prism 5 Software (La Jolla, CA, USA). Immunohistochemical data were analyzed by One way Anova test (group effect) and Mann–Whitney test (*p* < 0.0005).

## Results

### Clinical evaluation

The OP group included 48 patients with fragility hip fracture, T-score ≤ −2.5 SD and K–L score from 0 to 1. The OA group included 55 patients with radiographic evidence of hip OA with a K–L score 3 or 4 and T-score ≥ −2.5 SD (Table 2). There was no discrepancy for age, sex and comorbidities in the two groups (women, mean age OP 73 ± 3.2y OA 71 ± 4.1y) (Table 2). No significant differences were found in BMI values between the two groups (BMI mean values: OP, 26.59 ± 0.78 kg/m^2^; OA, 26.59 ± 0.78 kg/m^2^). CTRL patients were characterized by a T-score ≥ −1.0 SD and K–L score from 0 to 1.

**Table 2:** Main characteristics of OA, OP and CTRL patients

	OA	OP	CTRL	*T* test
				(Mann–Whitney test)
Age	71.00 ± 4.01	73.00 ± 3.20	44.23 ± 2.77	OP vs OA ns (P = 0.11); OP vs CTRL *** (P < 0.001); OA vs CTRL *** (P < 0.001)
BMI (kg/m^2^)	26.59 ± 0.78	25,61 ± 1.13	–	ns (P = 0.4467)
T score (L1–L4)	−0.71 ± 0.4	−2.85 ± 0.15	0.95 ± 0.01	OA vs OP *** (P < 0.0001)
T score (neck)	−0.64 ± 0.11	−2.77 ± 0.19	0.19 ± 0.05	*** (P = 0.0005)

Asterisks represent the significance level of the performed test (*P < 0.05, **P < 0.01, ***P < 0.001)

### Morphometric examination

**Fig. 1:**
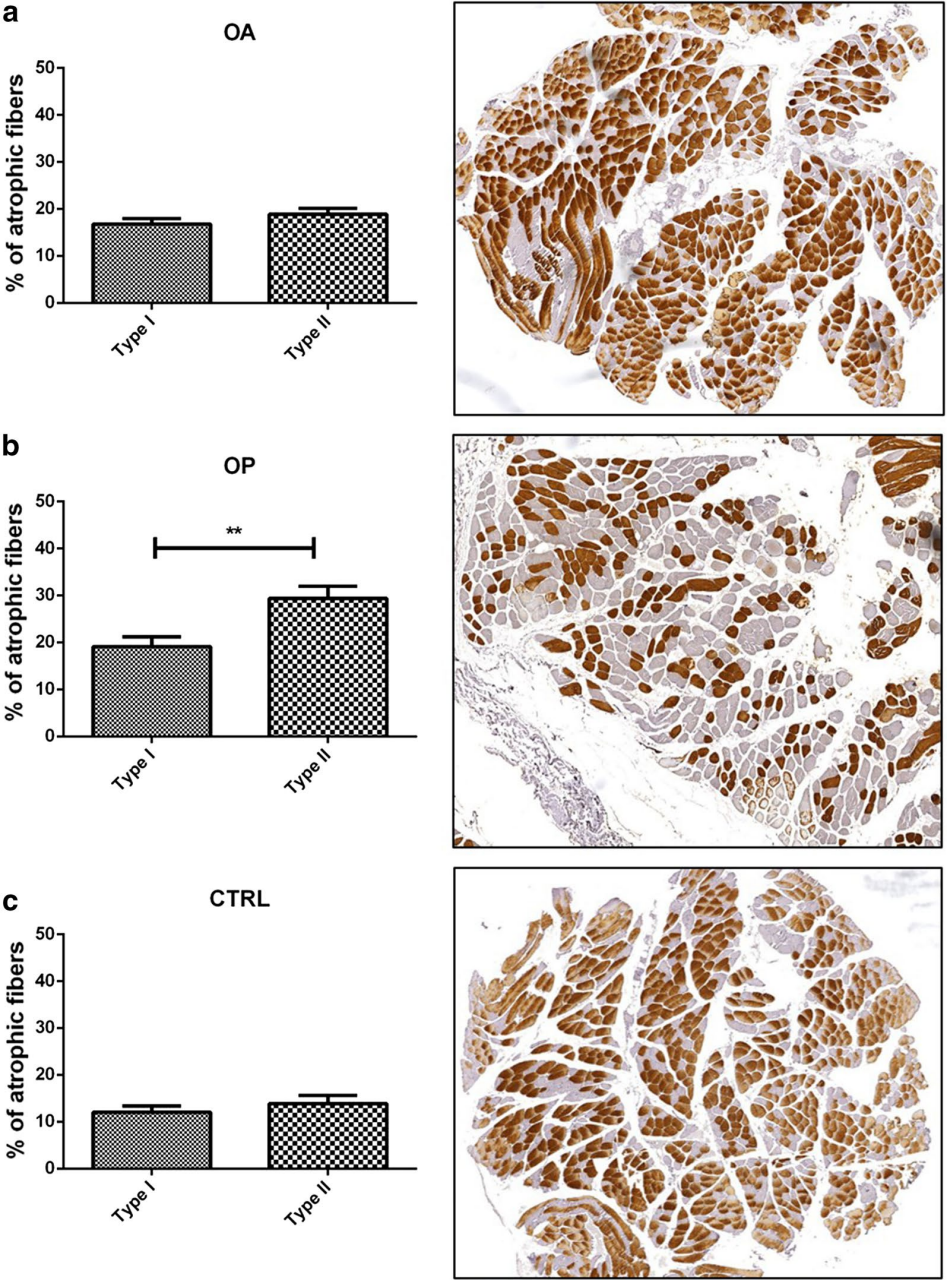
Mophometric analysis. Graphs show the percentage of muscle atrophic fibers in OA (**a**), OP (**b**) and CTRL (**c**) patients. Immunohistochemical analysis display type II fibers in OA (**a**), OP (**b**) and CTRL (**c**) group

Slow myosin antibody and fast myosin antibody stains allowed us to discriminate type I and type II fibers, respectively (Fig. 1). As already described [7, 11–13], OA and OP patients showed different pattern of fibers atrophy. In particular, OA patients showed about 30.00% of atrophic fibers with a diameter of less than 30 μm (16.81 ± 1.21% type I and 18.90 ± 1.24% type II) (Fig. 1a), whereas in OP group, we observed about 50.00% of atrophic fibers with prevalence of type II fibers (19.13 ± 2.07% type I and 29.41 ± 2.56% type II) (Fig. 1b). Control patients showed a percentage of atrophic fibers less than 15% (Fig. 1c). No significant difference was observed between genders.

### Immunohistochemical analysis of satellite cells

**Fig. 2:**
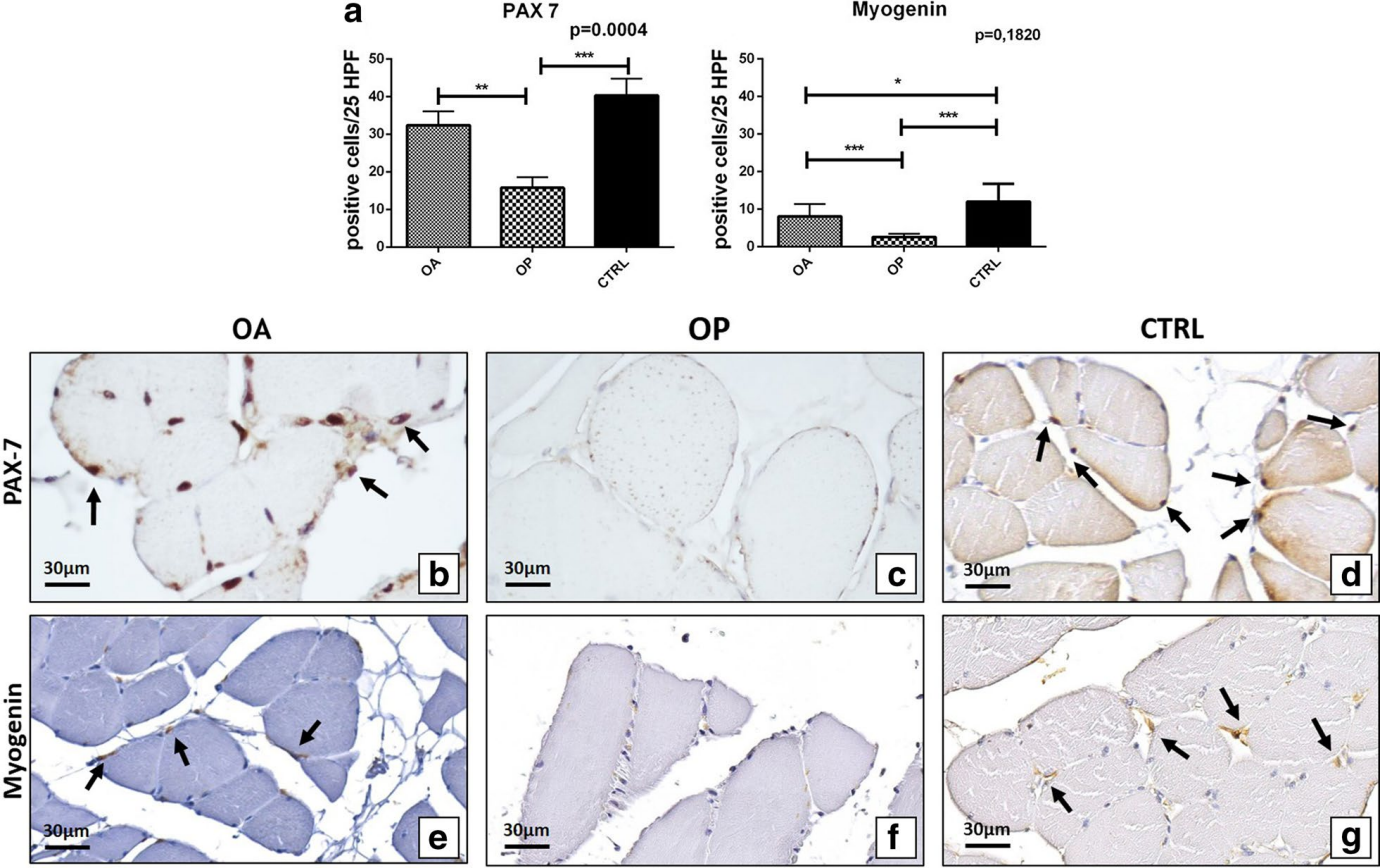
Satellite cells study. **a** Graph display the number of Pax7 and myogenin satellite cells in OA, OP and CTRL patients. **b** *Image* shows numerous Pax7 positive cells in a muscle biopsy of OA patient (*arrows*) (40×). **c** Muscle tissue of OP patient characterize by no/rare Pax7 positive satellite cells (40×). **d** Image displays numerous Pax7 positive cells in a muscle biopsy of a CTRL patient (*arrows*) (40×). **e** *Arrows mark* myogenin positive cells in a muscle biopsy of OA patient (40×). **f** Muscle tissue of OP patient characterize by no/rare myogenin positive satellite cells (40×). **g** Image displays numerous myogenin positive satellite cells in a muscle biopsy of a CTRL patient (*arrows*) (40×)

Immunohistochemistry results of Pax7 and myogenin expression were quantified by counting the number of positive satellite cells in 25 High Power Field (HPF) of randomly selected regions (Fig. 2). Our results showed a decrease of the number of both Pax7 and myogenin positive satellite cells in OP patients as compared to both OA and CTRL (Fig. 2a). In particular, Mann–Whitney test displayed a greater significant difference for the number of Pax7 positive satellite cells (OA 32.40 ± 3.70, OP 15.75 ± 2.85 p = 0.0075) (Fig. 2a–c) respect to myogenin (OA 8.12 ± 3.26, OP 2.55 ± 0.90 p = 0.036) (Fig. 2a, e, f). As aspect, under 50 patients (CTRL) showed higher number of Pax7 (40.33 ± 4.42) and myogenin (12.01 ± 4.71) positive satellite cells respect to both OP and OA groups (Fig. 2a, d, g). One way Anova test showed a significant group effect on the expression of Pax7 (p = 0.0004) (Fig. 2a).

### Ultrastructural characterization of satellite niches

**Fig. 3:**
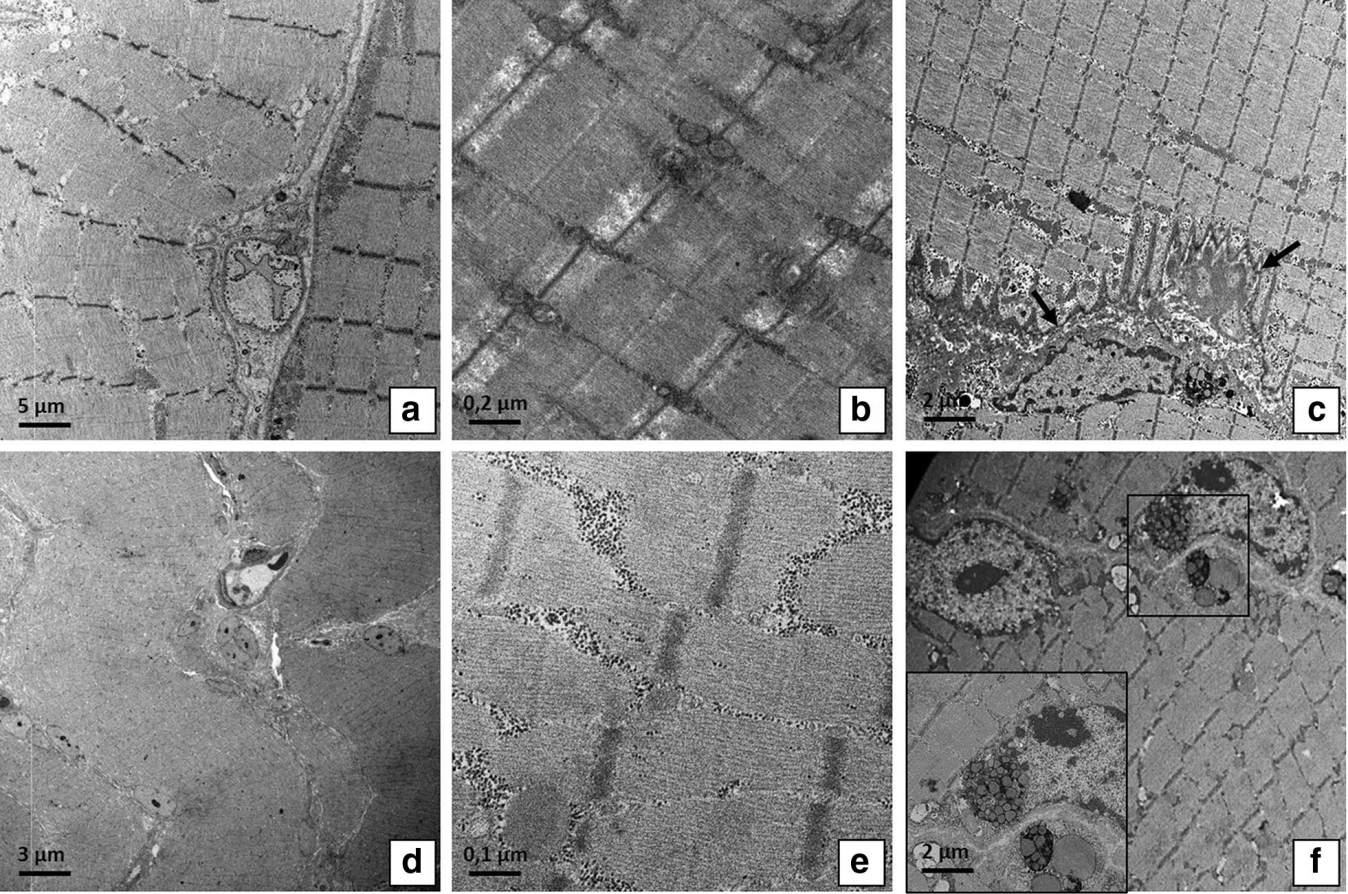
Ultrastructural analysis of muscle fiber and satellite cell niches. **a** *Image* shows muscle fibers of OA patient (2500×). **b** Well conserved sarcomere structure of a muscle fibers of OA patient (20,000). **c** Image displays satellite cells (*arrows*) strongly associated among them and fused to form a syncytium (5000×). **d** *Image* shows atrophic fibers in OP patient (1000×). **e** Misaligned sarcomere in muscle tissue of OP patient (20,000×). **f** Satellite cells with obvious mark of degeneration (*square*) (5000×)

TEM analysis was performed to characterize satellite cells niches and their cell syncytium. We found well conserved sarcomere ultrastructure and numerous satellite cells strongly associated among them or fused to form a syncytium, in OA muscle biopsies (Fig. 3a–c). Conversely, in OP patients we observed numerous atrophic fibers and rare satellite cells with obvious mark of degeneration (Fig. 3d–f).

### Immunohistochemical analysis of muscle fibers

**Fig. 4:**
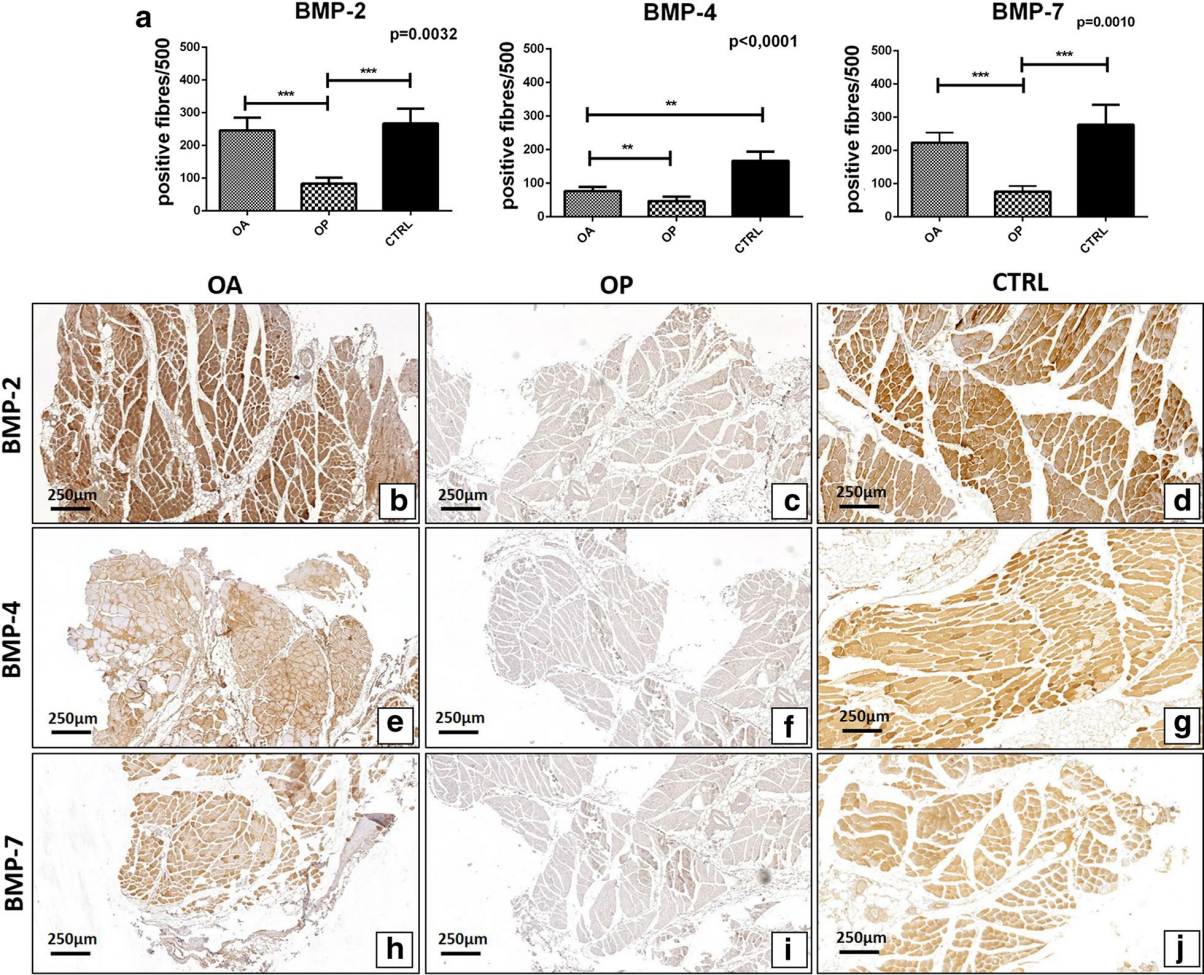
Immunohistochemical analysis of BMP2/4-7 expression. **a** Graph display the number of BMP2/4-7 muscle fibers in OA, OP and CTRL patients. **b** *Image* shows a muscle biopsy of OA patient with numerous BMP-2 positive fibers (4×). **c** Rare BMP-2 positive fibers in a muscle tissue of OP patient (4×). **d** Numerous BMP-2 positive fibers in a muscle biopsy of CTRL patient (4×). **e** Immunohistochemical reaction shows several BMP-4 positive fiber in a muscle biopsy of OA patient (4×). **f** *Image* shows a muscle biopsy of OP patient with rare/no BMP-4 positive fibers (4×). **g** Muscle of a CTRL patient characterize by high expression of BMP4 (4×). **h** Several BMP-7 positive fibers in a muscle tissue of OA patient (4×). **i** Immunohistochemical reaction shows no/rare BMP-7 positive fiber in a muscle biopsy of OP patient (4×). **j**, **d** Numerous BMP-7 positive fibers in a muscle biopsy of CTRL patient (4×)

**Fig. 5:**
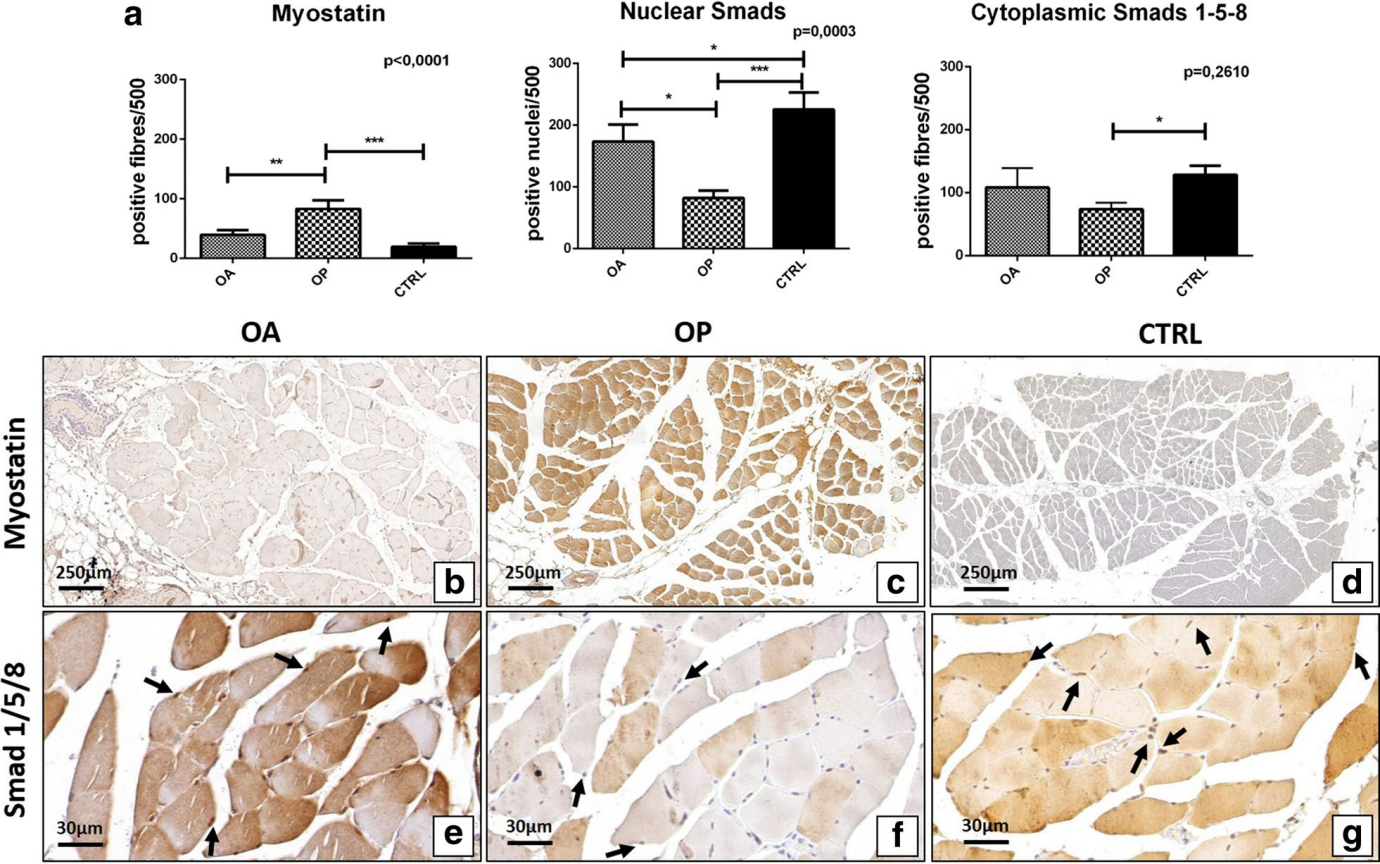
Analysis of the expression of myostatin and phosphorylated Smad1-5-8. **a** Graph display the expression of myostatin and phosphorylated Smad1-5-8 in muscle biopsies of OA, OP and CTRL patients. **b** Rare myostatin positive fibers in a muscle tissue of OA patient (4×). **c** Muscle tissue of OP patient characterizes by numerous myostatin positive fibers (4×). **d** Muscle of a CTRL patient characterize by no expression of myostatin (4×). **e** *Image* shows muscle tissue of OA patient with numerous phosphorylated Smad1-5-8 positive nuclei (*arrows*) (40×). **f** Muscle of OP patient with phosphorylated Smad1-5-8 negative nuclei (*arrows*) (40×). **g** Image displays muscle biopsy of CTRL patient with numerous phosphorylated Smad1-5-8 positive nuclei (*arrows*)

Immunohistochemistry results of BMP-2, BMP-4, BMP-7, myostatin and Smads1-5-8 were quantified by counting the number of positive fibers (out of a total of 500 in randomly selected regions). As concern Smads, we also evaluated the number of positive nuclei (out of a total of 500 in randomly selected regions). A significant group effect was detected for BMP-2 (p = 0.0032), BMP-4 (p < 0.0001), BMP-7 (p = 0.0010) (Fig. 4a), myostatin (p < 0.001) and nuclear Smad1-5-8 (p = 0.0003) (Fig. 5a). No significant group effect was found for the cytoplasmatic expression of Smad1-5-8 (p = 0.2610) (Fig. 4a). Mann–Whitney test revealed significant differences of BMP-2 expression in OA group as compared to OP (OA 245.4 ± 39.21, OP 82.55 ± 19.01 p < 0.0001) (Fig. 4a–c). In particular, immunohistochemistry displayed an intense cytoplasmic reaction for BMP-2 close to regenerating fibers of OA patients (Fig. 4b). In accordance to what was observed in terms of BMP-2, we found a significant difference on the rate of BMP-4 and BMP-7 (Fig. 4a). For BMP-4, Mann–Whitney test showed significant differences in OA group respect to OP (OA 76.57 ± 12.07, OP 46.17 ± 13.00, p = 0,0015) (Fig. 4a, e, f). In addition, we also observed significant difference between OA and CTRL groups (p = 0.0020) (Fig. 4a). Likewise, a significant difference in OA group respect to OP were found for BMP-7 expression (OA 223.70 ± 30.44, OP 76.16 ± 17.16, p < 0.0001) (Fig. 4a, h, i). We detected an increase in fibers showing myostatin staining in OP patients as compared to OA (OA 39.36 ± 7.96, OP 83.13 ± 14.46, p = 0.0028) (Fig. 5a–c). As concern cytoplasmic staining of phosphorylated Smad1-5-8, no significant differences were observed between OA and OP patients (OA 101.90 ± 15.22, OP 137.50 ± 22.92, p = 0.3106) (Fig. 5a, e, f). Conversely, Mann–Whitney test displayed a significant difference in the nuclear expression of phosphorylated Smad1-5-8 in OA group as compared to OP patients (OA 173.10 ± 27.91, OP 81.90 ± 12.03, p = 0.0136) (Fig. 5a, e, f). Significant difference was also observed between OA and CTRL group (p = 0.0258) and OP and CTRL patients (p < 0.0001) (Fig. 5).

## Discussions

Sarcopenia is one of the most important age-related human diseases. It is characterized by the loss of muscle mass, strength and function. Recent studies reported a strong association between sarcopenia and the occurrence of degenerative bone diseases such as osteoporosis [6]. Then, the bone-muscle crosstalk could be considered a key element to understand the patho-physiogenesis of sarcopenia. In this context, our and other studies demonstrated the role of BMPs in satellite cells activity and in the onset of sarcopenia [8, 10–13].

The main aim of this study was to test the hypothesis that the balance between BMPs and myostatin pathways regulates the age-related muscle degeneration in OP and OA patients. To this end, we investigated the relationship among the expression of BMP-2/4-7, myostatin and phosphorylated Smads1-5-8 and the muscle quality, evaluated in term of fibers atrophy and satellite cells activity.

**Fig. 6:**
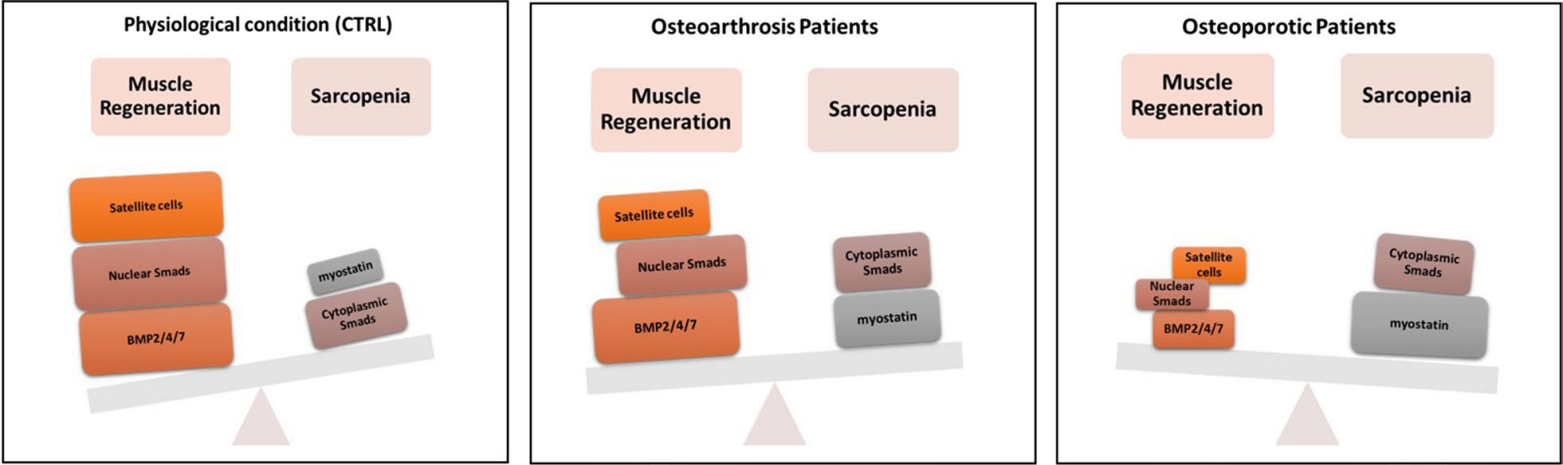
Cellular and molecular characteristics of Sarcopenia in CTRL, OA and OP patients

According to our previous studies [7, 11], morphometric analysis showed an increase of the number of atrophic fibers in OP patients compared to OA. In particular, muscle degeneration was mainly related to type II fibers atrophy in OP patients. In line with these data, we found an high regenerative potential in muscle tissues of OA patients due to the significant amount of Pax7 positive satellite cells detected in OA group. Satellite cells play an indispensable role in muscle regeneration. The self-renewing proliferation of these cells not only maintains the stem cell population but also provides numerous myogenic cells, which proliferate, differentiate, fuse, and lead to new myofiber formation and reconstitution of a functional contractile apparatus [25]. In addition, residual satellite cells of OP patients displayed signs of degeneration in niche, mitochondria and nucleus ultrastructural. The loss of satellite cells, and/or their degeneration, could reflect the alteration of muscle metabolism that occurs in patients affect by osteoporosis. Recently, Sartori et al. highlights the role of BMPs in muscle metabolism. In particular, authors demonstrated the ability of some BMPs (mainly the BMP-2) to induce satellite cells activation and myofibers formation [8, 10]. Moreover, BMP-2 and BMP-4 can stimulate the differentiation of muscle satellite cells in mature osteoblasts during fracture healing process [26]. In muscle environment, BMPs are produced by muscle fibers, endothelial cells and, eventually, by inflammatory cells [27]. It is known that BMP signaling, acting through Smad1, Smad5 and Smad8 (Smad1-5-8) and Smad-4, regulates muscle regeneration in mouse models. Indeed, the inhibition of BMP signaling causes muscle atrophy, abolishes the hypertrophic phenotype of myostatin-deficient mice and strongly exacerbates the effects of denervation and fasting [10]. Recently, we investigated the expression of BMP-2 and -4 in a small cohort of human muscle biopsies demonstrating an inverse correlation between their expression and muscle atrophy [11–13]. Here, we finalized the BMPs study investigating the expression of the most important BMPs involved in muscle metabolism, (BMP2/4 and -7), in a population of 123 patients. Our data clearly indicated the decrease of BMP2/4 and -7 expression in OP patients compared to both OA group and CTRL. Noteworthy, we observed a remarkable difference in the expression of BMP-2 and -7 respect to BMP-4 in OA patients. In particular, the expression of BMP-2 and 7 was threefold increase compared to BMP-4. This data suggest that BMP-4 could play a minor role in muscle homeostasis of adults. To circumstantiate the influence of BMPs signaling in sarcopenia occurrence, we also investigated the expression of myostatin. It is a powerful negative regulator of muscle growth [18, 19]. The signaling triggered by the binding between myostatin and type II Ser/Thr kinase receptor induces the phosphorylation of Smad-2 and -3 (R-Smads) and the formation of R-Smad/Smad-4 complexes [18]. These complexes translocate to the nucleus where they regulate the transcription of target genes involved in muscle homeostasis [18]. Then, the signaling of myostatin negatively influences the BMPs pathways sequestering Smad-4 [8]. Our results showed that myostatin expression is strongly dependent to both aging and bone diseases. Indeed, we found higher levels of myostatin in OP respect to OA patients (same age range). A different expression profile was also found for phosphorylated Smad1-5-8 between OP and OA patients In particular, OP patients showed a low number of positive nuclei. The localization of Smad1-5-8 in muscle fibers allowed us to obtained information about the balance between BMPs and myostatin signaling. Indeed, in presence of both myostatin and BMPs expression it is possible to observe an increase of cytoplasmic phosphorylated Smad1-5-8, whereas in samples characterized by no/low expression of myostatin and high expression of BMPs, phosphorylated Smad1-5-8 are mainly localized into the nucleus (Fig. 6). All together, our data displayed a specific molecular and histological profile of sarcopenic muscle tissues of OP and OA patients. In particular, muscle of OP patients are characterized by (a) high number of atrophic fibers, (b) low number of both quiescent and activated satellite cells and (c) an unbalance between BMPs and myostatin signaling (Fig. 6). Conversely, muscle tissue of OA patients showed cellular and molecular characteristics similar to the CTRL group (Fig. 6).

## Conclusions

The identification of molecular pathways involved in the pathogenesis of sarcopenia open new prospective for the development of drugs able to prevent/treat the muscle impairment that occur in elderly. Results here reported, highlighting the role of BMPs and myostatin pathways in physio-pathogenesis of human sarcopenia, allow us to propose human recombinant BMP-2/7 and anti-myostatin antibodies as a possible therapeutic option for sarcopenic patients.

### Limits of the study

A possible limit of the present study could be represented by the control group. Indeed, the control patients showed significant age difference to the OP and OA groups (44 vs 73–71). Unfortunately, it is very difficult to collect muscle biopsies of patients over 70 without OP or OA who underwent hip arthroplasty for high-energy hip fractures. However, we believe that the data obtained from the control group are valid, and can improve the understanding of the role of BMPs and myostatin pathways in in muscle homeostasis. Another limit of the present study could be represented by the different timing in which we performed DXA analysis on OP and OA patients. In particular, DXA exam was performed one day before surgery for OA patients and one month after surgery OP patients (according to our operational guidelines). Difference in the timing of the DXA exam could influence the obtained BMD values. Nevertheless, these possible discrepancies do not influence the diagnosis of osteoporosis given the occurrence of a low-energy femoral fracture.

## Abbreviations

BMD: bone mineral density
BMI: body mass index
DAB: 3,3′ Diaminobenzidine
DXA: dual-energy X-ray absorptiometry
H&E: hematoxylin and eosin
HPF: high power field
HRP: Horseradish peroxidase
JSN: joint space narrowing
K–L: Kellgren–Lawrence
OA: osteoarthritis
OP: osteoporosis
PAX7: paired box protein
SMAD: small mother against decapentaplegic
TEM: transmission electron microscopy
TGF-β: transforming growth factor beta
